# Supplementary material for: Splice-Junction-Based Mapping of Alternative Isoforms in the Human Proteome
Source: Cell Rep. Author manuscript; Available in PMC 2020 Jan 15. (PMC6961840; doi:10.1016/j.celrep.2019.11.026)
Supplement: 3 [file NIHMS1546469-supplement-3.zip › DF2/PXD0010154/Liver-519-P49757-VVAPQSPTFQGTEWGQSSGAASPGLFQAGHR.pdf]

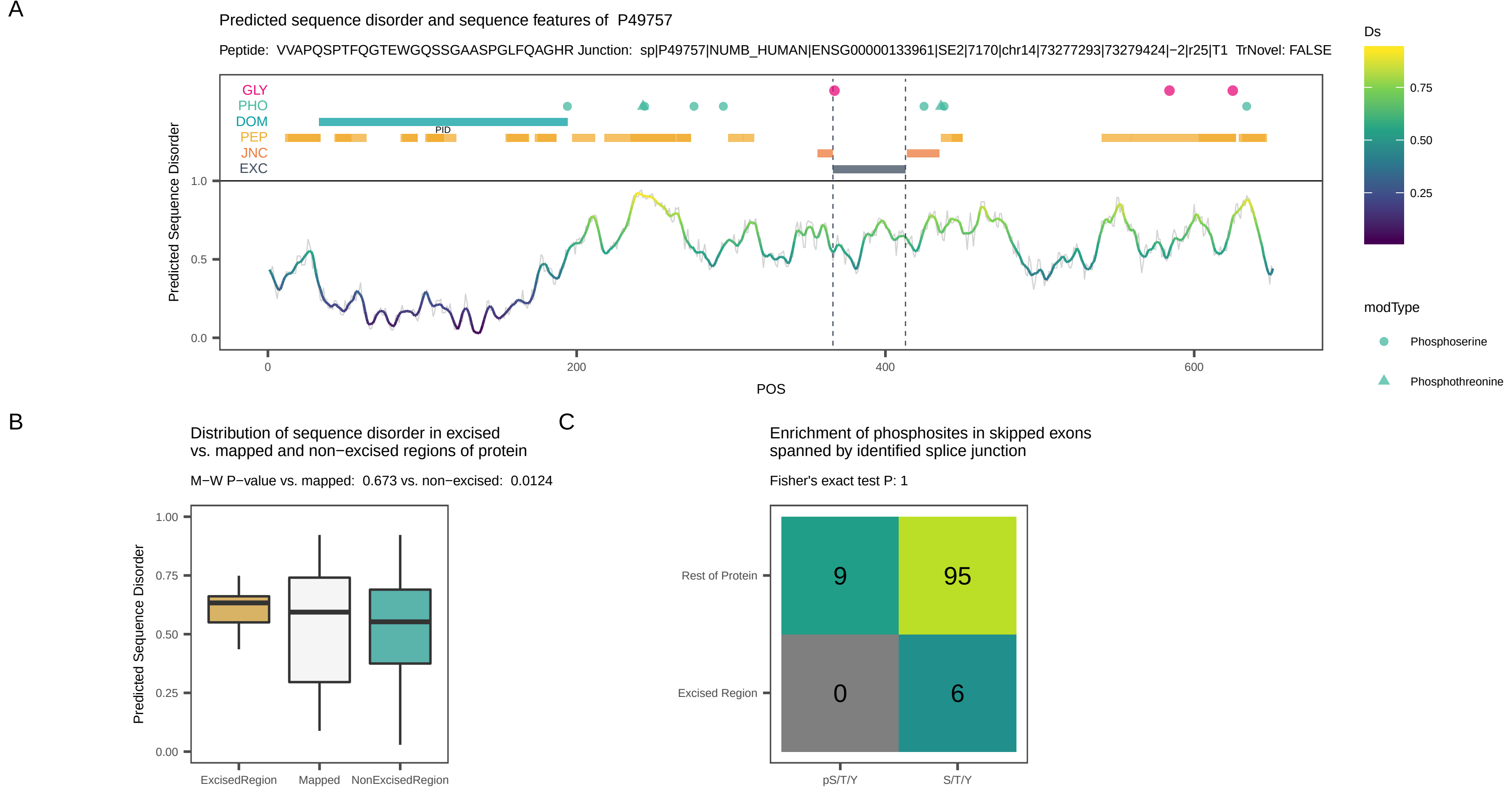
A
Predicted sequence disorder and sequence features of P49757
Peptide: VVAPQSPTFQGTEWGQSSGAASPGLFQAGHR Junction: sp|P49757|NUMB_HUMAN|ENSG00000133961|SE2|7170|chr14|73277293|73279424|−2|r25|T1 TrNovel: FALSE
GLY
PHO
DOM
PEP
JNC
EXC
PID
Predicted Sequence Disorder
1.0
0.5
0.0
0
200
400
600
POS
Ds
0.75
0.50
0.25
modType
Phosphoserine
Phosphothreonine
B
Distribution of sequence disorder in excised
vs. mapped and non−excised regions of protein
M−W P−value vs. mapped: 0.673 vs. non−excised: 0.0124
Predicted Sequence Disorder
1.00
0.75
0.50
0.25
0.00
ExcisedRegion
Mapped
NonExcisedRegion
C
Enrichment of phosphosites in skipped exons
spanned by identified splice junction
Fisher's exact test P: 1
Rest of Protein
Excised Region
9
95
0
6
pS/T/Y
S/T/Y
